# Giant pandas can discriminate the emotions of human facial pictures

**DOI:** 10.1038/s41598-017-08789-y

**Published:** 2017-08-16

**Authors:** Youxu Li, Qiang Dai, Rong Hou, Zhihe Zhang, Peng Chen, Rui Xue, Feifei Feng, Chao Chen, Jiabin Liu, Xiaodong Gu, Zejun Zhang, Dunwu Qi

**Affiliations:** 1 Sichuan Key Laboratory of Conservation Biology for Endangered Wildlife, Chengdu Research Base of Giant Panda Breeding, Chengdu, Sichuan 610081 China; 2 Key Laboratory of Southwest China Wildlife Resources Conservation, China West Normal University, Nanchong, Sichuan 637009 China; 3 Chengdu Institute of Biology, Chinese Academy of Sciences, Chengdu, Sichuan 610041 China; 4 Wildlife Conservation Division, Forestry Department of Sichuan Province, Chengdu, Sichuan 610000 China

## Abstract

Previous studies have shown that giant pandas (*Ailuropoda melanoleuca*) can discriminate face-like shapes, but little is known about their cognitive ability with respect to the emotional expressions of humans. We tested whether adult giant pandas can discriminate expressions from pictures of half of a face and found that pandas can learn to discriminate between angry and happy expressions based on global information from the whole face. Young adult pandas (5–7 years old) learned to discriminate expressions more quickly than older individuals (8–16 years old), but no significant differences were found between females and males. These results suggest that young adult giant pandas are better at discriminating emotional expressions of humans. We showed for the first time that the giant panda, can discriminate the facial expressions of humans. Our results can also be valuable for the daily care and management of captive giant pandas.

## Introduction

Animals adjust their behaviour based on their information-recognition ability^1–3^. Some types of recognition are innate, but others are acquired through learning (acquisition)^4,5^. Emotional facial expressions serve critical adaptive purposes^6^. Emotional facial expressions evolved for physiological functions^7,8^ and conspecific interactions^9–11^, and it is also involved in interspecific interactions^12^. Recent research has shown that domestic animals can discriminate human facial expressions^13,14^. Some research has also demonstrated that captive wildlife can recognize human faces^15^, while it is still unknown whether non-primate wildlife can discriminate the emotional facial expressions of humans.

Giant pandas have been shown to maintain their social relationships through visual as well as chemical (e.g., glandular secretions or urine) communication^16–19^. Giant pandas exhibit marking behaviour by peeling bark or disturbing the soil with their claws and directly transfer visual information through body language^20,21^. In addition, pandas can discriminate between black-and-white objects with only subtle differences in shape, implying they can perception face-like stimuli^22^. However, previous research has shown that giant pandas cannot recognize themselves in a mirror but instead consider the image to be a separate conspecific individual, indicating they do not have the capacity of self-recognition^23^. Therefore, many questions remain about the cognitive abilities of giant pandas.

Giant pandas have been bred in captivity for only 64 years and have never been intentionally selected and bred for panda–human interactions^24^. Understanding the ability of the giant panda as a wild animal to discriminate human facial expressions can provide valuable information for their daily care. In addition to their close interaction with keepers due to their daily care^24^, nearly three million tourists visit the Chengdu Research Base of Giant Panda Breeding to see the approximately one hundred and fifty giant pandas and ten new cubs at the facility each year. Thus, these giant pandas are exposed to the various expressions of a large number of visitors each day. Since giant pandas have visual and cognitive abilities, their response to human contact may lead to changes in behaviour and recognition, as has been found in domestic dogs (*Canis familiaris*)^25^ and cats (*Felis catus*)^26^ as well as birds^27^.

The captive breeding of giant pandas has been so successful that the captive population of giant pandas reached 471 in 2016^28^. Subsequently, a program was launched to release captive giant pandas into the wild to reinforce wild populations. For captive-bred giant pandas, constant contact with humans is inevitable during pre-release training, which is essential for their survival in the wild^29^.

In this paper, following the methods of Müller *et al*.^30^, we aimed to examine the ability of pandas to discriminate different emotion of human faces. A previous study showed that giant pandas can recognize face-like geometric patterns^22^. To rule out the possibility that giant pandas can discriminate among facial expressions simply based on the geometric relationships among facial features, we presented giant pandas with pictures of halves of faces with happy or angry expression and tested whether they can choose correct stimuli.

## Results

Eighteen adult giant pandas, nine females and nine males, were assigned to 4 groups based on the rewarded stimulus (happy or angry faces) and horizontal facial part (upper or lower half of the face) prior to the experiment (Table 1). To familiarize the subjects with the facial discrimination tasks and to select cooperative individuals for follow-up tests, pictures of faces with neutral expressions and the back of the head were simultaneously presented to the giant pandas in the pre-training phase. Ten (6 females and 4 males) giant pandas showed the ability to discriminate pictures of the face from those of the back of the head and entered the first stage trial (Table 1). There was no significant difference in age between the giant pandas that passed the pre-training and those that failed (Fig. S1, *t*-test, *t*_16_ = 0.170, *P* = 0.887), and there was also no difference in the number of males and females (Table S1, chi-square test: χ^2^_1_ = 0.900, *P* = 0.343).

**Table 1:** Subjects, experimental groups and training performances.

Name	Studbook no.	Sex	Age (years)	Pre-training: no. sessions^a^	First stage set	Rewarded expression	First stage: no. sessions^a^
A Bao	801	Female	5	**27**	Upper	Happy	**10**
Qi Fu	709	Female	7	**10**	Lower	Happy	**30**
Xing Rong	680	Female	8	5	Lower	Happy	NA
Qi Zhen	490	Female	16	**15**	Lower	Happy	24
Shu Qing	480	Female	16	NA	Upper	Happy	NA
Yong Bing	738	Male	7	**15**	Lower	Happy	**20**
Xi Lan	731	Male	7	30	Lower	Happy	NA
Mei Lan	649	Male	9	10	Upper	Happy	NA
Bing Dian	520	Male	15	**28**	Upper	Happy	33
Mei Bing	737	Female	7	**16**	Upper	Angry	**15**
Bei Chuan	785	Female	7	**6**	Upper	Angry	**8**
Da Jiao	845	Female	7	30	Upper	Angry	NA
Xing Ya	881	Female	8	**13**	Lower	Angry	35
Wu Yi	830	Male	7	5	Upper	Angry	NA
Xing Bing	814	Male	10	12	Upper	Angry	NA
Qiao	824	Male	11	NA	Lower	Angry	NA
Xiong Bing	540	Male	14	**24**	Lower	Angry	31
Qiu Bing	574	Male	12	**10**	Lower	Angry	**30**

N/A: not applicable. ^a^Bold type indicates that the learning criterion was reached.

**Figure 1:**
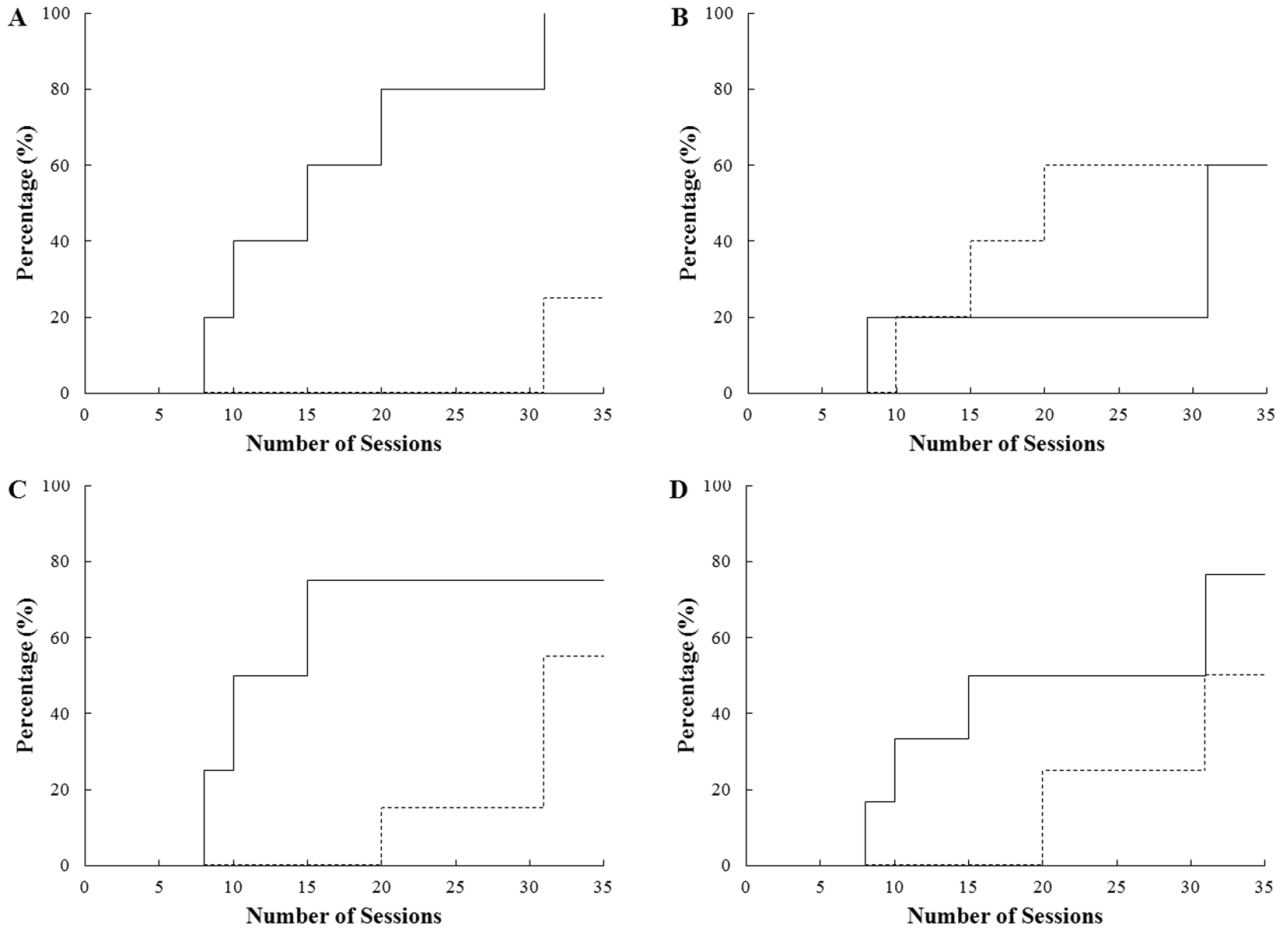
The survival curve of the giant pandas meeting the first-stage criterion. (**A**) Cumulative proportion of young giant pandas (solid line) and older giant pandas (dashed line) that reached the criterion. (**B**) Cumulative proportion of giant pandas that reached the criterion in the angry group (solid line) and the happy group (dashed). (**C**) Cumulative proportion of giant pandas that reached the criterion when shown the upper face (solid line) and the lower face (dashed line). (**D**) Cumulative proportion of female giant pandas (solid line) and male giant pandas (dashed line) that reached the criterion.

All giant pandas that entered the first stage were simultaneously presented with two horizontal half-face pictures from the same person, one happy and one angry. The stimulus showing the happy expression was the rewarded stimulus for the happy group (5 individuals), whereas for the other five in the angry group, the stimulus with the angry expression was rewarded (Table S2). A total of 5 young adult giant pandas (5–7 years old) and 1 older individual (older than or equal to 8 years) met the requirement (≥70% correct choices) within 30 sessions in the first stage (similar to Nagasawa *et al*.^25^). Another 2 male and 2 female individuals, with ages ranging from 8 to 16 years old, failed in this stage (Table 1). Analysis of the Cox proportional hazards model showed that the young adult pandas reached the learning requirement at a significantly faster rate than the older ones (Fig. 1A; proportional hazards model: n = 10, z = 2.285, *P* = 0.02).

Three giant pandas from the happy group and three from the angry group achieved the learning criterion of the first stage. Analysis of the Cox proportional hazards model showed that there was no significant difference in the speed of achieving the criterion between the happy and angry groups (Fig. 1B; proportional hazards model: n = 10, z = 0.49, *P* = 0.63). This indicated that the emotion associated with the human expression did not affect the learning speed of the giant pandas. For the upper and lower face groups, three giant pandas that were shown the upper face and three that were shown the lower face met the requirement, and there was no significant difference in the learning rate between the two groups (Fig. 1C; proportional hazards model: n = 10, z = 1.29, *P* = 0.20). Additionally, there was no significant difference in the rate of learning between females and males (Fig. 1D; proportional hazards model: n = 10, z = 0.92, *P* = 0.36).

**Figure 2:**
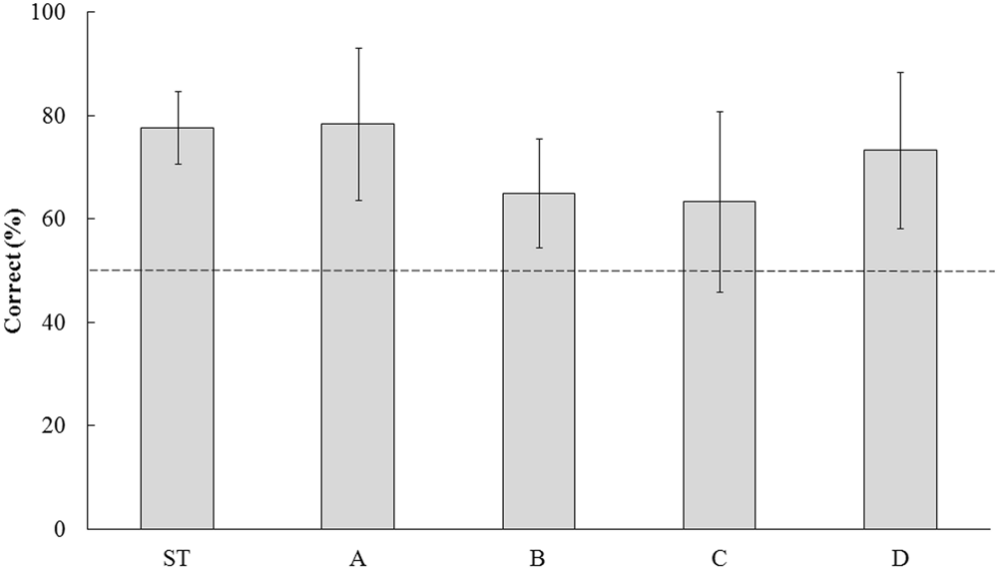
Ratios of correct responses in the standard trials (ST) and the four types of probe trials in the second stage. Proportion of conditioned correct choices for the 5 subjects in the second stage in standard trials (150 trials per subject) and in each type of probe trial (10 trials per subject): (**A**) the same horizontal portion of the face as shown in the standard trials but with novel faces; (**B**): the other horizontal half of the same faces used in the standard trials; (**C**): the other horizontal half of the novel faces; (**D**): the left half (vertical) of the same faces used in the standard trials.

In the second stage, standard trials and probe trials were carried out on 6 giant pandas that passed the first stage (Table S3). The standard trials were employed to reinforce the behaviour of expressions selection. Four types of probe trials were carried out to test whether giant pandas can discriminate facial expressions based on facial features rather than simply their memory of the pictures as well as to test whether giant pandas can do so based on the features of the entire face rather than local features. The other halves of the faces from the same persons as the first stage were used as stimuli in the probe trials, so did pictures of same halves and the other halves of novel faces. We expected that giant pandas can use global information of whole face by linking the information from one horizontal half faces to the other horizontal half, if they can choose the same emotion as the first stage^30^. All six giant pandas performed significantly better than chance in all of the probe trials (Table 2, Fig. 2). However, there were no significant differences among the probe trials in terms of the proportion of correct choices (Fig. 2; generalized linear mixed model: χ^2^_3_ = 3.225, *P* = 0.358), and we found that the proportion of correct choices did not differ between females (n = 4) and males (n = 2) (Fig. S2; generalized linear mixed model: *F*_1,223_ = 0.191, *P* = 0.663) or between the angry (n = 3) and happy (n = 3) groups (Table S3; generalized linear mixed model: *F*_1,223_ = 0.152, *P* = 0.697) in the second stage.

**Table 2:** Binomial models comparing performance in the four types of probe trials and the level of significance.

Probe trial	Estimate*	Standard error	z_59_	*P*
a	1.285	0.313	4.101	<0.001
b	0.619	0.271	2.287	0.022
c	0.547	0.268	2.040	0.041
d	1.012	0.292	3.465	0.001

^a^The same horizontal half of the face as in the standard trials but with novel faces; b: the other horizontal half of the same faces used in the standard trials; c: the other horizontal half of the novel faces; d: the left half (vertical) of the faces of the same persons used in the standard trials.

^*^Coefficient estimate of binomial models with logit link, value of zero indicates a choice probability of 50%.

## Discussion

This study revealed that giant panda can discriminate human emotional expressions based on information from the entire face rather than local features when they are presented with pictures of half of a face. This ability to discriminate human emotional expressions has been identified in various species, especially domestic animals^31–34^, and dogs^30^ and pigeons (*Columba livia*)^31,33,35^ can do this based on information from the entire face. Note that the experimenter holding the pictures was not blind to the stimuli in this study. We can not, therefore, rule out the possibility that giant pandas can get unintentional hint from the experimenter (i.e. Clever Hans effect), though we tried to avoid it.

Our results showed that a negative stimulus (angry expression) did not affect the learning ability of the giant panda. An angry expression, which is generally accompanied by a threat or punishment, can have strong effects on captive or domestic animals^32–35^. A previous study found that dogs show better learning ability when rewarded for touching a happy stimulus than when rewarded for touching an angry stimulus^30^. A physiological reaction was also recorded in horses when they were exposed to angry facial expressions^36^. Our results did not show an effect of the valence of the stimuli (happy versus angry) on the speed of learning. This was possibly because the giant pandas used in this study have never experienced any negative treatment, such as threats or punishment, due to the rules and regulations for feeding and management^24^, and they therefore cannot associate negative experiences with angry expressions. However, without detailed and extensive experiments on the effects of angry expression, we can not rule out the possibility that angry expression can exert other behavioral or physiological impact on giant pandas. Further studies are also needed to explore whether the emotions of keepers and visitors can affect giant pandas.

In this study, young adult giant pandas (5–7 years old) exhibited a better learning ability than older individuals (8–16 years old). The learning abilities of animals change over their lifetime, increasing rapidly from infancy to the young adult stage; then, depending on the specific ability, learning ability either improves, is maintained, or declines in old age^37,38^. Studies have revealed that wild giant pandas learn most of their survival skills before 1.5 years of age, when they live with their mothers as cubs^20^. Wild subadult giant pandas have been reported to learn mating behaviours by watching adults during the mating season^39^.

As captive-bred giant pandas are now being released into the wild to supplement the small wild populations, enhancing the survival ability of captive-bred giant pandas before release is essential, especially in the early stage^29,40^. Our results suggest that young giant pandas are more suitable for pre-release training than old ones. We also suggest selecting young individuals for release because learning is necessary to adapt to the wild environment; for example, it allows potential predators to be identified or suitable habitat to be found.

Gender differences in learning ability vary among species^25,41–43^, but females tend to show superior learning abilities over males^44–47^. Females dogs respond more obviously than males do to human emotions^48,49^. In this study, however, we did not find a significant difference in the ability to discriminate human facial expressions between female and male giant pandas. Note that our results are limited, since “negative” findings from the trials may be a result of small sample sizes^50^.

Only pictures of male humans were used as stimuli to avoid introducing an extra factor of stimulus gender, given the small sample size of giant pandas in this study. Stimulus gender can affect the learning behaviour of animals^43^. Nagasawa *et al*.^25^ found that the ability of dogs to discriminate facial expressions increased when they were shown faces of the same gender as their owner rather than those of the opposite gender^25^. It will be interesting to test whether giant pandas can discriminate the facial expressions of female humans as well as those of males.

## Methods

### Animals and treatments

A total of 18 adult giant pandas at the Chengdu Research Base of Giant Panda Breeding were selected as subjects; information about the pandas is presented in Table 1. The 9 females and 9 males were in good health during the research phase, which began in September 2015 and ended in February 2016. All experimental procedures were approved by the Animal Care and Use Committee of the Chengdu Research Base of Giant Panda Breeding and were performed in accordance with its guidelines.

**Figure 3:**
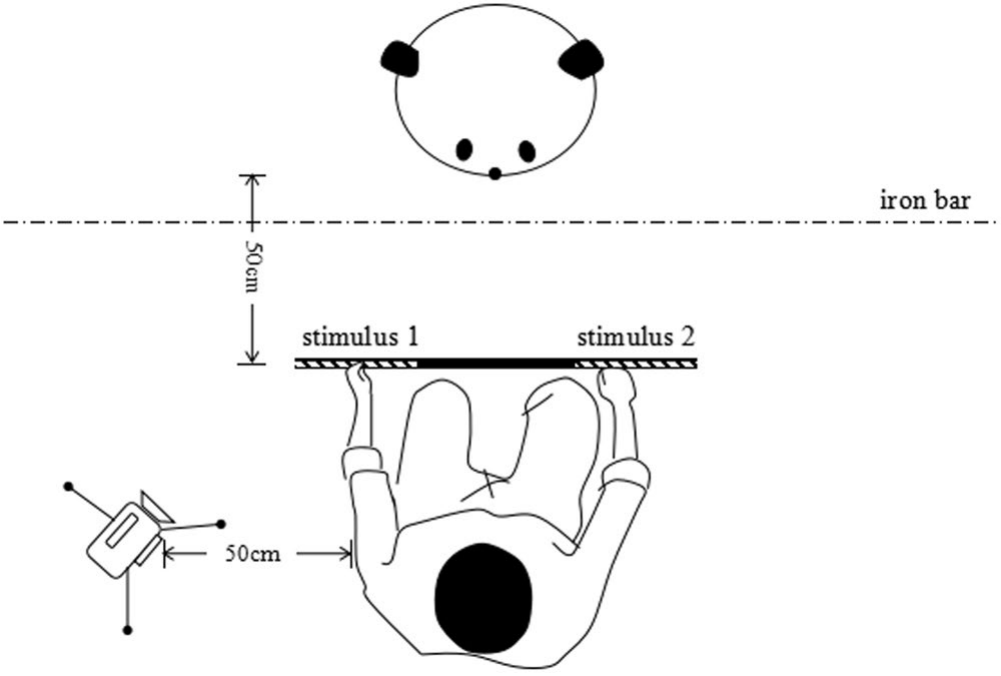
Overhead sketch of the experimental setup (see text for details). This diagram was created using Microsoft Paint, https://support.microsoft.com.

All individuals were divided into groups by horizontal half-face classes and expression classes in both the first stage and second stage. For class of horizontal half-face, we had upper- and lower-face groups; the images of the faces were divided at the horizontal midline of the nose (Fig. 3). There were 9 subjects each in the upper-face and lower-face groups. The class of facial expression consisted of 2 groups, the happy group and the angry group, and each group had 9 individuals (Table 1). In the happy group, pictures of happy human faces (stimuli) were shown as correct stimuli, and in the angry group, pictures of humans with angry expressions were shown as correct stimuli.

### Experimental apparatus

The experiment was conducted in different pens at the Chengdu Research Base of Giant Panda Breeding, generally from 8:00 to 11:00 and from 14:00 to 16:00; only one panda was tested at a time. The pens were approximately 4 × 5 m, and the lighting was similar to that under natural conditions. The giant pandas were attracted to the iron bars by calling their names, and they tended to sit near or hold the iron bars. The response of captive giant pandas to calling has been established during the subadult stage to facilitate their daily care. The experimenter squatted and held a transparent plastic sheet (65 × 25 cm) facing the panda within a distance of 50 cm; the sheet was placed at a height that allowed the subject to visually perceive the stimuli and indicate a choice with its nose. The experimenter’s line of sight was focused on giant pandas, and avoid potential unintentional hint on choices. A SONY FDR-AXP35 camera (Sony Corporation, Konan Minato-ku, Tokyo, Japan) was placed 50 cm behind the experimenter and to the left to record the tests (Fig. 3).

**Figure 4:**
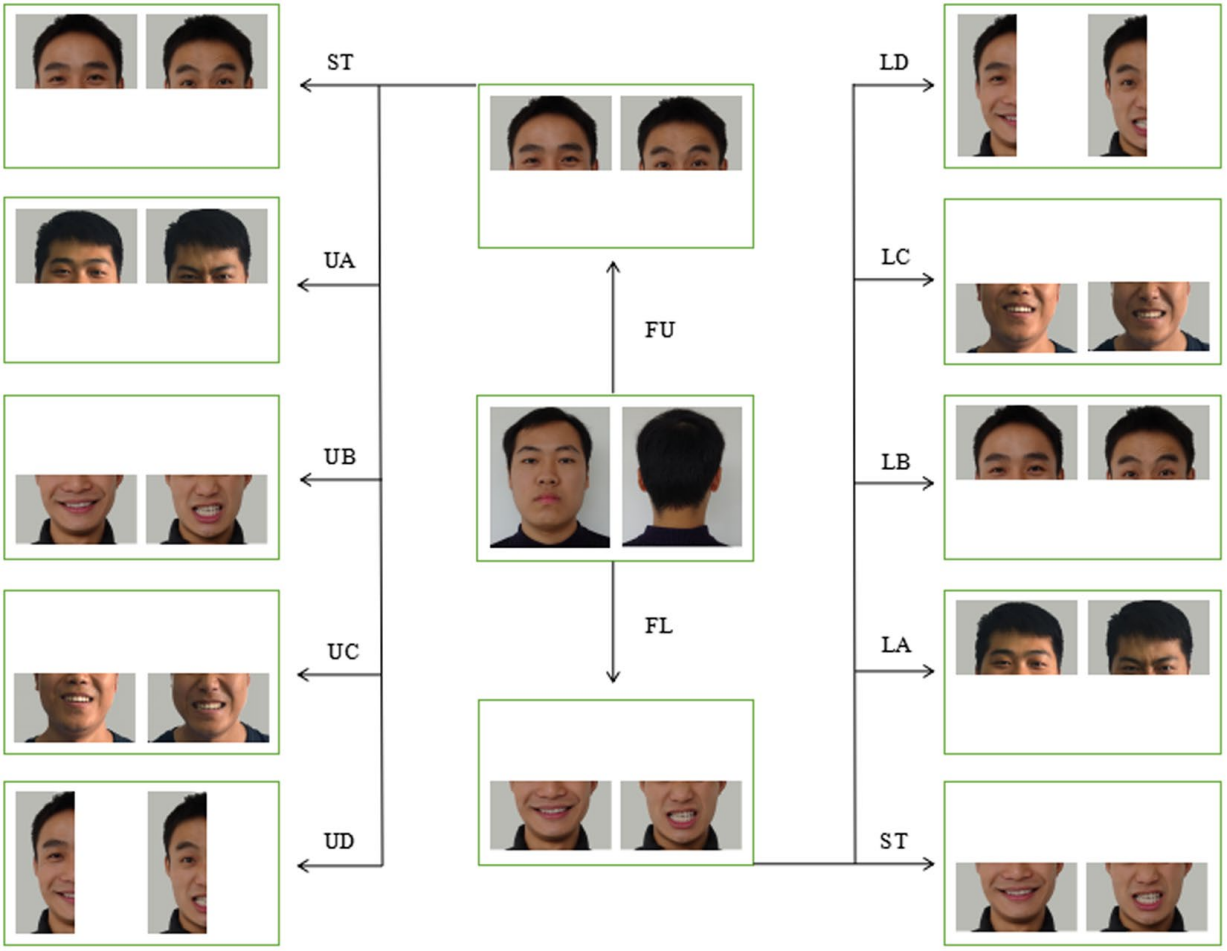
Some pictures used as stimulus pairs in this study. All pictures were of the faces of adult Chinese men, and the pre-training stimuli and test stimuli were pictures of members of our laboratory. Shown are example stimulus pairs in the pre-training set and the two first-stage sets as well as example stimulus pairs in the probe trials for a subject trained with the upper or lower halves of the faces (left and right columns, respectively). All picture pairs have been reproduced with the permission of the depicted person. FU: first stage trial in upper group; FL: first stage trial in lower group; ST: standard trial; UA: same portion, novel face in upper group; UB: other horizontal half, same face in upper group; UC: other horizontal half, novel face in upper group; UD: left half (vertical), same face in upper group; LA: same horizontal portion, novel face in lower group; LB: other horizontal half, same face in lower group; LC: other horizontal half, novel face in lower group; LD: left half (vertical), same face in lower group.

Two pictures (stimuli) were placed directly on the transparent plastic sheet and positioned 20 cm away from each other (Fig. 4). Seventeen pairs of pictures were presented as A4-size pictures (25.4 cm × 20.3 cm). The faces used in the pictures were of young men with short and black hair, without any associated accessories (such as glasses or a hat), with a gray background. Posed expressions were captured, following a guild from Ekman and Friesen^51^. Every stimulus was taken 5 times, and the most typical ones were chosen for experiment based on the agreement of three authors of this manuscript. The pictures of faces were split at the middle of the nose horizontally or vertically depending on the design of the trial.

### Experimental procedure

We conducted a two-way discrimination experiment in which the selection of either of the two stimuli was consistently rewarded with food (apple pieces) and conditioned with a whistle. The subjects were trained to indicate one stimulus with their noses in two ways. 1) The subject shifted its head and touched the iron bars with the tip of the nose pointing in the direction of the target stimulus. 2) The subject’s face was pressed to the iron bars in the direction of the target stimulus or the target stimulus was touched with the tip of the nose. Either of these two behaviours indicated that the subject had completed one trial. The subjects underwent sessions consisting of 30 trials each day. One trial was no more than 50 s with breaks of between 5 and 30 s separating the trials, and each session lasted between 2 and 15 min. If a subject choose neither of the stimuli after a break, the experiment continued on a different day. To eliminate any confounding effects of human gender, only pictures of men were used in this study.

The experiment consisted of pre-training and two stages of discrimination training that differed only in the stimuli presented. In each stand-alone trial, the same stimuli (pictures of the same person) were shown on the left or right side of the sheet. The two pictures shown to giant panda are also from a same person in each trial. In each session, the frequencies of left and right placement of the stimuli were equal, but they were presented in a random sequence. If the subject touched the correct stimulus based on the conditions defined above, the experimenter immediately provided a small slice of apple and whistled for 1 s. If the subject indicated the incorrect stimulus, no food reward was given and no sound was played until the subject chose the correct stimulus.

### Pre-training

The aim of pre-training was to familiarize the pandas with the discrimination tasks and to select cooperative individuals for follow-up tests. Pictures of two keepers were used as stimuli. A pair of pictures of the same person, one of the face and one of the back of the head, was shown to subjects in each trial, and subjects were rewarded for touching the face stimulus. The placement (left or right) of the face stimulus was randomly chosen before the trials. In total, 30 sessions of trials were carried out, and each session included 30 trials. Pictures of both keeper were used 15 times in each session in a random sequence.

A subject passed a session if it selected the correct stimulus 24 times in 30 trials, and 3 consecutive passed sessions were required to achieve our success criteria (similar to Müller *et al*.^30^). The subjects that met the criteria advanced to the next stage, and the others were removed from the experiment.

### First stage

The aim of the first stage was to test the different learning rates of the pandas and their reactions to emotion. Eighteen subjects were assigned to groups based on the upper/lower half (horizontal) of faces and happy/angry expression (Table S2). One group (n = 9) was shown only the upper halves of faces (upper group), and the other group (n = 9) was shown only the lower halves of faces (lower group). A pair of pictures, one happy and one angry, was shown to each giant panda in a trial. The subjects were rewarded for touching the happy stimulus or angry stimulus depending on the group to which they were assigned. The pictures of 10 strangers were used in this stage. Each session included 30 trials, and each stranger’s pictures were used in 3 trials. The position of the rewarded stimulus and the sequence of strangers’ pictures were randomized.

A giant panda passed a session if it selected the correct stimulus 21 times in 30 trials (corresponding to p < 0.05, binomial test), and 4 passed sessions out of any consecutive 5 sessions were required to achieve our success criteria. The subjects that met the criteria advanced to the next stage, and those that failed within 30 sessions were removed from the experiment.

### Second stage

The aim of the second stage was to explore whether the pandas could recognize the emotional expressions of human faces or simply used local cues for discrimination. The same procedure used for group assignment in the first stage was used (Table S3). Two trial types were carried out in this stage, i.e., standard trial and probe trial. The standard trial followed the same procedure as in the first stage using the same pictures (old pictures) as stimuli and rewards depending on the group assignment. Four types of probe trials (Fig. 4) were designed: a. pictures of novel faces, same horizontal half; b. the other horizontal half of the old pictures; c. the other horizontal half of the novel faces; and d. the left half (vertical) of the old pictures. A total of 5 pairs of novel pictures of unfamiliar people (different from those in the first stage) were used in the probe trials. Only the left halves of the faces were used in probe trial d to rule out the possibility of lateral gaze bias^52,53^.

In this stage, the four probe trials were conducted between every third standard trial in alphabetical order. Each session comprised 15 standard trials and 4 probe trials (19 trials), and each subject underwent a total of 10 sessions (190 trials). In the probe trials, the subjects were rewarded for selecting either happy or angry faces, but whether the selection was correct, depending on the group assignment, was recorded.

### Statistical analyses

We compared the rate at which the giant pandas met the success requirement between groups using Cox proportional hazards models in the “survival” package^54^. Performance in the four types of probe trials was compared using a generalized linear mixed effects model (GLMM) in the package “lme4”^55^, assuming a binomial distribution with a log-link function. The IDs of the giant pandas were included as random factors to account for the repeated measures structure of the dataset. The proportions of correct choices to the tests under the four experimental conditions were compared to a 50% level of chance using binomial generalized linear models (GLMs). All analyses were performed in the R 3.32 environment^56^.

## Electronic supplementary material

Supplementary Information
