# Author Correction: Giant pandas can discriminate the emotions of human facial pictures

**DOI:** 10.1038/s41598-018-21907-8

**Published:** 2018-03-01

**Authors:** Youxu Li, Qiang Dai, Rong Hou, Zhihe Zhang, Peng Chen, Rui Xue, Feifei Feng, Chao Chen, Jiabin Liu, Xiaodong Gu, Zejun Zhang, Dunwu Qi

**Affiliations:** 1 Sichuan Key Laboratory of Conservation Biology for Endangered Wildlife, Chengdu Research Base of Giant Panda Breeding, Chengdu, Sichuan 610081 China; 2 Key Laboratory of Southwest China Wildlife Resources Conservation, China West Normal University, Nanchong, Sichuan 637009 China; 3 Chengdu Institute of Biology, Chinese Academy of Sciences, Chengdu, Sichuan 610041 China; 4 Wildlife Conservation Division, Forestry Department of Sichuan Province, Chengdu, Sichuan 610000 China

Correction to: *Scientific Reports* 10.1038/s41598-017-08789-y, published online 16 August 2017

This Article contains an error in the Methods section under subheading ‘Animals and treatments’.

“All experimental procedures were approved by the Animal Care and Use Committee of the Chengdu Research Base of Giant Panda Breeding and were performed in accordance with its guidelines.”

should read:

“All experimental procedures were approved by the Research Center and the Animal Husbandry Department of the Chengdu Research Base of Giant Panda Breeding and were performed in accordance with their guidelines and regulations.”

