# Supplementary material for: Giant pandas can discriminate the emotions of human facial pictures
Source: Sci Rep. 2017 Aug 16;7:8437. doi: 10.1038/s41598-017-08789-y (PMC5559457; doi:10.1038/s41598-017-08789-y)
Supplement: Supplementary file 1 — Supplementary Information [file 41598_2017_8789_MOESM1_ESM.doc]

**Giant pandas can discriminate the emotions of human facial pictures**

Youxu Li^a,b,*^, Qiang Dai^c,*^, Rong Hou^a^, Zhihe Zhang^a^, Peng Chen^a^, Rui Xue^a^, Feifei Feng^a^, Chao Chen^a^, Jiabin Liu^a^, Xiaodong Gu^d^, Zejun Zhang^b^ & Dunwu Qi^a^

^a^ Sichuan Key Laboratory of Conservation Biology for Endangered Wildlife, Chengdu Research Base of Giant Panda Breeding, Chengdu, Sichuan 610081, China

^b^ Key Laboratory of Southwest China Wildlife Resources Conservation, China West Normal University, Nanchong, Sichuan 637009, China

^c^ Chengdu Institute of Biology, Chinese Academy of Sciences, Chengdu, Sichuan 610041, China

^d^ Wildlife Conservation Division, Forestry Department of Sichuan Province, Chengdu, Sichuan 610000, China

^*^ These authors contributed equally to this work.

Correspondence and requests for materials should be addressed to D.Q. (Dunwu Qi, e-mail: [qidunwu@163.com](mailto:qidunwu@163.com)) or Z.Z. (Zejun Zhang, e-mail: [zhangzj@ioz.ac.cn](mailto:zhangzj@ioz.ac.cn))

**Appendix: Details of experiment and results of test**

## Pre-training

**Stimuli:** face and back of head

**Reward:** face

**Subjects:** 18 giant pandas (female:9, male:9)

**Results:**


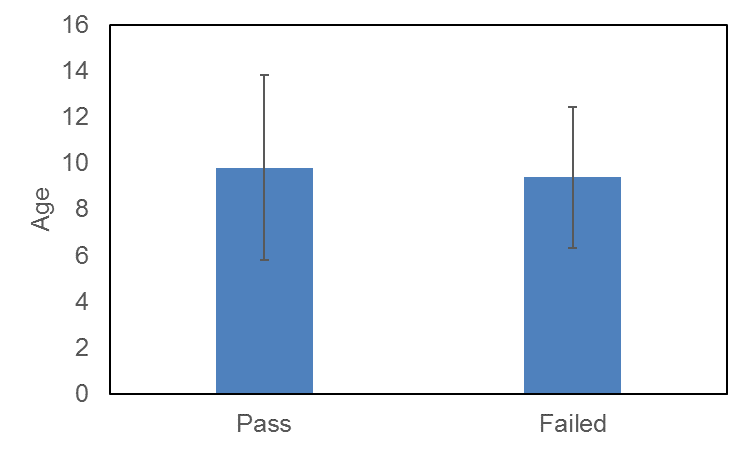


Figure S1. Average age of giant pandas that passed and failed to pass the pre-training. Error bars show the +/- 1.0 standard deviation.

Table S1. The gender of giant pandas that passed and failed to pass the pre-training

|  | Pass | Fail |
| --- | --- | --- |
| Female | 6 | 3 |
| Male | 4 | 5 |

## The first stage test

**Stimuli:** happy and angry facial expression pictures

**Reward:**

happy group: happy facial expression pictures

angry group: angry facial expression pictures

**Subjects:** 10 giant pandas (6 females and 4 males)

**Group:**

Table S2. Group assignment in the first stage test

|  | **Upper face** | **Lower face** | **Sum** |
| --- | --- | --- | --- |
| **Happy group** | 1 female, 1 male | 2 females, 1 male | 3 females, 2 males |
| **Angry group** | 2 females, 1 male | 1 female, 1 male | 3 females, 2 males |
| **Sum** | 3 females, 2 males | 3 females, 2 males | 6 females, 4 males |

## The second stage test

**Stimuli:** happy and angry facial expression pictures

Standard trial: pictures used in the first stage

Probe trail: pictures used in the first stage and pictures of novel faces

**Reward:**

Standard trial: happy or angry facial expression pictures, depending on group assignment, similar to the first stage.

Probe trail: both correct and wrong choices were rewarded.

**Subjects:** 6 giant pandas (4 females and 2 males)

**Group:**

**Table S3. Group assignment in the second stage test**

|  | **Upper face** | **Lower face** | **Sum** |
| --- | --- | --- | --- |
| **Happy group** | 1 female, 0 male | 1 females, 1 male | 2 females; 1 male |
| **Angry group** | 2 females, 0 male | 0 female, 1 male | 2 females; 1male |
| **Sum** | 3 females, 0 males | 1 females, 2 males | 4 females, 2 males |


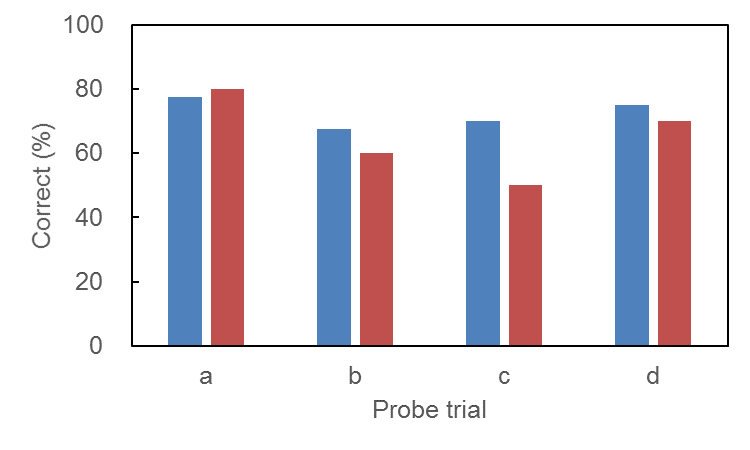


Figure S2. Average percentage of correct selections by female (blue) and male (red) pandas in the probe trials.


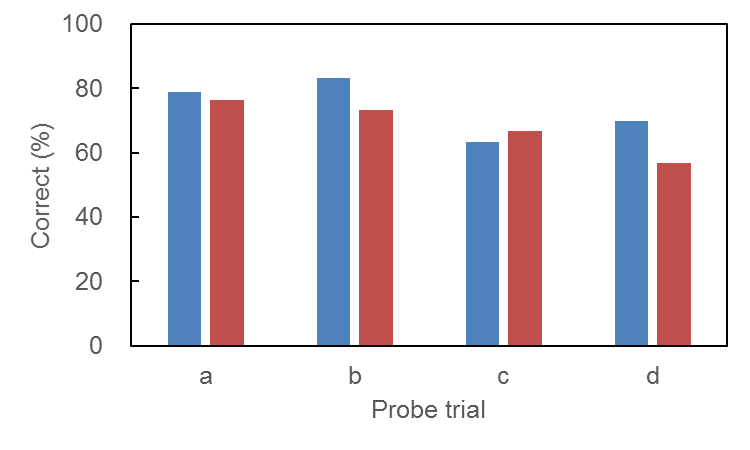


Figure S3. Average percentage of correct selections by giant pandas in the happy group (blue) and in the angry group (red) in the probe trials.
